# Transcriptional and functional consequences of *TP53* splice mutations in colorectal cancer

**DOI:** 10.1038/s41389-019-0141-3

**Published:** 2019-05-15

**Authors:** Jørgen Smeby, Anita Sveen, Ina A. Eilertsen, Stine A. Danielsen, Andreas M. Hoff, Peter W. Eide, Bjarne Johannessen, Merete Hektoen, Rolf I. Skotheim, Marianne G. Guren, Arild Nesbakken, Ragnhild A. Lothe

**Affiliations:** 1 Department of Molecular Oncology, Institute for Cancer Research, Oslo University Hospital, Oslo, Norway; 2 K.G. Jebsen Colorectal Cancer Research Centre, Division of Cancer Medicine, Oslo University Hospital, Oslo, Norway; 3 Department of Oncology, Oslo University Hospital, Oslo, Norway; 4 Institute of Clinical Medicine, Faculty of Medicine, University of Oslo, Oslo, Norway; 5 Department of Gastroenterological Surgery, Oslo University Hospital, Oslo, Norway

**Keywords:** Colorectal cancer, Cancer genetics

## Abstract

*TP53* mutations are common in colorectal cancer (CRC). Most *TP53* sequencing studies have been restricted to coding regions, but recent studies have revealed that splice mutations can generate transcript variants with distinct tumorigenic and prognostic properties. Here, we performed unrestricted sequencing of all coding sequences and splice regions of *TP53* in a single-hospital series of 401 primary CRCs. *TP53* splice mutations were detected in 4% of the cases (*N* = 16), considerably more frequent than reported in major databases, and they were mutually exclusive to exon mutations. RNA sequencing revealed high-level expression of aberrant transcript variants in the majority of splice mutated tumors (75%). Most variants were predicted to produce truncated TP53 proteins, including one sample expressing the potentially oncogenic and druggable p53ψ isoform. Despite heterogeneous transcript structures, downstream transcriptional profiling revealed that *TP53* splice mutations had similar effects on TP53 target gene expression and pathway activity as exonic mutations. Intriguingly, *TP53* splice mutations were associated with worse 5-year relapse-free survival in stage II disease, compared to both *TP53* wild-type and exon mutations (*P* = 0.007). These data highlight the importance of including splice regions when examining the biological and clinical consequences of *TP53* mutations in CRC.

## Introduction

Mutations in the tumor suppressor gene *TP53* have long been known to be integral to colorectal carcinogenesis^1,2^, substantiated by its high mutation rate in manifest colorectal cancers (CRC)^3^. The *TP53* mutation-spectrum and distribution show a large proportion of missense mutations clustering on hotspot codons, while truncating alterations, including nonsense, frameshift and splice site mutations, constitute ~20–25% of the mutations. Specifically, mutations at canonical splice sites account for approximately 2% of all detected *TP53* mutations, according to major databases^4,5^. However, this may be underestimated since mutation analyses have been restricted to coding regions and the DNA-binding domain in many studies.

The consequences of *TP53* splice mutations on the expression of the corresponding transcript variants are diverse, with smaller studies providing evidence of alternative splicing by exon skipping, intron retention, generation of novel splice sites and usage of cryptic splice sites^6^. On the other hand, the effects of *TP53* splice mutations on downstream target gene expression and pathway activity have not been elucidated. Intriguingly, some truncating *TP53* mutations have been shown to produce transcriptionally inactive isoforms leading to activation of a prometastatic cellular program^7,8^. These separation-of-function isoforms possess distinct oncogenic properties with potentially prognostic and therapeutic relevance.

In a single-hospital series of 401 primary CRCs, we investigated the downstream consequences of splice mutations in *TP53*, including expression of aberrant transcript variants, target genes, pathway activity and potential clinical consequences.

## Results

### The spectrum of coding and splice mutations in *TP53*

**Fig. 1:**
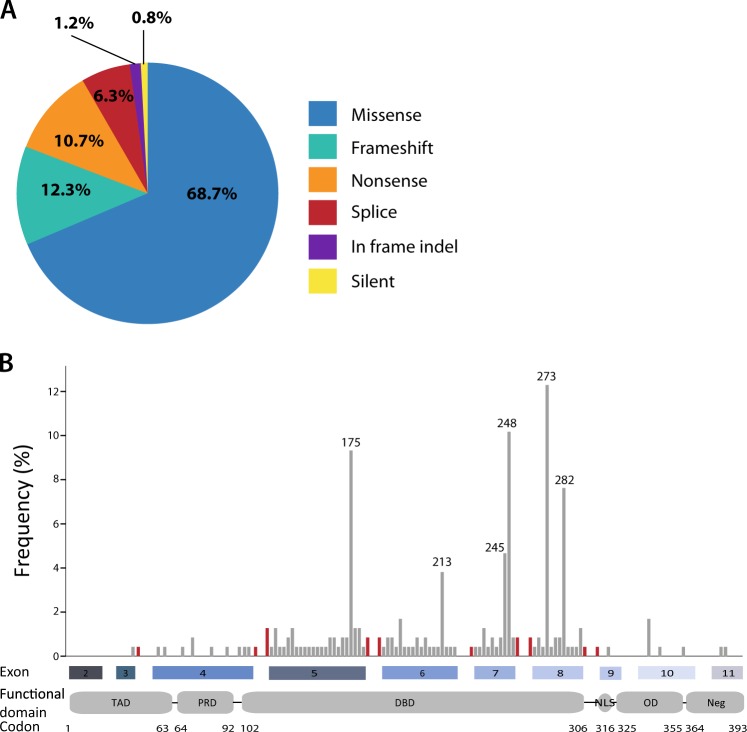
*TP53* mutation spectrum in primary colorectal cancers. **a** Proportion of *TP53* mutation types, plotted as the percentage of the total number of detected mutations (*N* = 252). **b** Frequency and distribution of *TP53* splice (red) and exon (gray) mutations at each position along the gene (codon number is indicated below). The frequency is calculated relative to all mutations included in this plot (*N* = 236; exon mutations encompassing multiple codons, i.e. indels, are excluded). Frequency of splice mutations represents all mutations affecting the relevant splice region; see Table 1 for details. TAD transactivation domain, PRD proline-rich domain, DBD DNA-binding domain, NLS nuclear localization domain, OD oligomerization domain, Neg negative-regulation domain

*TP53* was mutated in 60% (*N* *=* 241) of the 401 cases, including 4% (*N* = 16) with splice mutations. The total number of mutations was 252, with double mutations in 2.7% (*N* = 11) of the tumors. Missense mutations were most common (69%, *N* *=* 173), while splice mutations accounted for 6% of all *TP53* mutations (Fig. 1a) and were more frequent than the corresponding 2% reported in the IARC *TP53* database^4^. Of the 16 *TP53* splice mutations, one had not been previously described in cancer, and nine were novel to CRC (according to the IARC *TP53* Database in April 2016; Supplementary Table S1)^4^. Thirteen were point mutations at the consensus splice sites, five in the donor site and eight in the acceptor site, of which 12 were single base substitutions and one was a single base deletion (Table 1). The three remaining splice mutations consisted of a point mutation located five basepairs downstream of exon 4, a duplication of six basepairs spanning the exon 7/intron 7 boundary and a deletion of 14 basepairs spanning the intron 5/exon 6 boundary. The majority of splice mutations (*N* = 13, 81%) was located adjacent to exons 5 to 8, reflecting the distribution of *TP53* exon mutations (Fig. 1b). Notably, all splice mutations were mutually exclusive to *TP53* exon mutations.

**Table 1:** Characteristics of *TP53* splice mutations and corresponding transcript variants

Sample no.^a^	*TP53* splice mutation	c_description^b^	Aberrant transcript variant(s)	Disturbed reading frame	Premature stop codon	Predicted protein product
1	Exon 3+1 (SD), G >A	c.96+1 G>A	Exon 3 skipping	Yes	Yes	Truncated
2	Exon 5–1 (SA), G >A	c.376–1 G>A	Cryptic SA exon 5	No	No	Loss of 7 AAs 5′ exon 5
3	Exon 7+1 (SD), G >A	c.782+1 G>A	Intron 7 retention	Yes	Yes	Truncated
4	Exon 5–1 (SA), G >A	c.376–1 G>A	Cryptic SA exon 5	No	No	Loss of 7 AAs 5′ exon 5
5	Exon 9–2 (SA), A >G	c.920–2 A>G	(I) Intron 8 retention (II) Exon 9 skipping	(I) No (II) Yes	(I) Yes (II) Yes	(I) Truncated (II) Truncated
6	Exon 8+1 (SD), G >A	c.919+1 G>A	(I) Intron 8 retention (II) Exon 8 skipping	(I) No (II) Yes	(I) Yes (II) Yes	(I) Truncated (II) Truncated
7	Exon 5–1 (SA), G>T	c.376–1 G>T	Cryptic SA exon 5	No	No	Loss of 7 AAs 5′ exon 5
8	Exon 8–1 (SA), G >T	c.783–1 G>T	(I) Intron 7 retention (II) Cryptic SA exon 8	(I) Yes (II) Yes	(I) Yes (II) No	(I) Truncated (II) Loss of 9 AA 5′ exon 8, disturbed reading frame
9	Exon 4+5, G >A	c.375+5 G>A	Intron 4 retention	No	Yes	Truncated
10	Exon 7–2 (SA), A >T	c.673–2 A>T	(I) Intron 6 retention	No	Yes	Truncated
			(II) Cryptic SA intron 6	Yes	Yes	Truncated
11	Exon 5+1 (SD), G >A	c.559+1 G>A	(I) Intron 5 retention (II) Cryptic SD exon 5	(I) Yes (II) Yes	(I) Yes (II) Yes	(I) Truncated (II) Truncated
12	Exon 6–1 (SA), G >A	c.560–1 G>A	(I) Intron 5 retention (II) Exon 6 skipping	(I) Yes (II) Yes	(I) Yes (II) Yes	(I) Truncated (II) Truncated
13	Exon 5+1 (SD), del G	c.559+1del1	Not present	N/A	N/A	Full length
14	Exon 6–9 (SA), del 14 bp	c.560–9_564del14	Not present	N/A	N/A	Full length
15	Exon 7+3, ins 6 bp	c.782+3_782+4ins6	Not present	N/A	N/A	Full length
16	Exon 8–2 (SA), A>G	c.783–2 A>G	Not present	N/A	N/A	Full length

*SD* consensus splice donor site, *SA* consensus splice acceptor site, *AA* amino acid

**Fig. 2:**
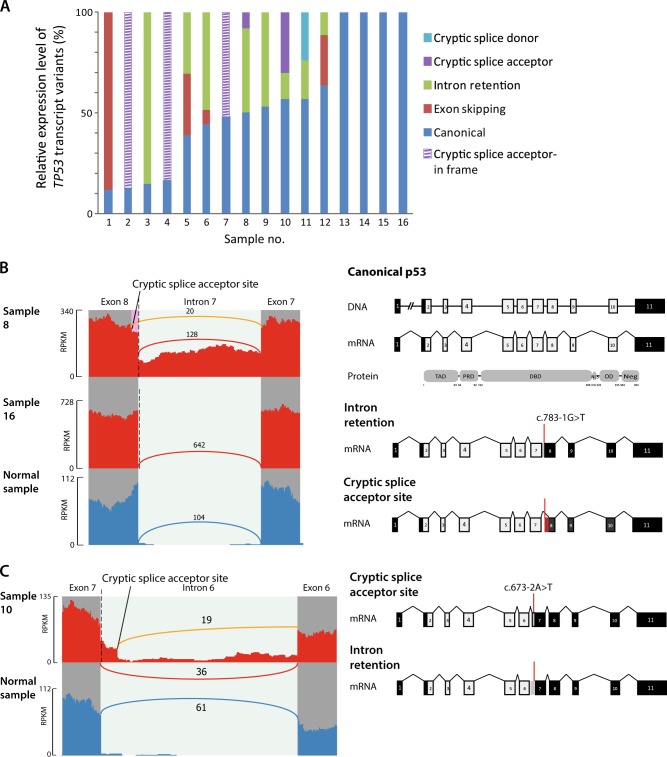
Transcript variants in *TP53* splice mutated colorectal cancers. **a** Estimated relative expression levels of *TP53* transcript variants in 16 splice mutated samples. Samples are ordered according to the relative expression level of the canonical splicing variant. Canonical splicing, cryptic splice sites and exon skipping events were quantified in the Sashimi plots, while intron retention values are the median depth of the relevant intronic region as measured by IRFinder. **b** Sashimi plots from two tumor samples with point mutation in the canonical splice acceptor site of intron 7 (marked with a dashed line) compared with a normal sample. Reads spanning exon junctions are represented by arcs, and each arc is labeled with the number of supporting reads. The arc representing an aberrant splicing event is colored in orange. Heights of bars reflect the read depth at each genomic position (reading frame right to left). Schematic visualization of the canonical *TP53* transcript variant is shown in the top panel, and the two aberrant variants caused by the splice site mutation below, with coding sequences in light gray and noncoding sequences in black. In sample 8, 20 junction reads span transcripts using a cryptic acceptor site located 24 basepairs into exon 8, while 106 reads retain intron 7 (median depth of intron 7 as measured by IRFinder). Contrastingly, in both sample 16 and the normal sample all junction reads between exon 7 and exon 8 span the canonical splice sites. The transcript variant retaining intron 7 is predicted to generate a premature stop codon. The usage of an alternative splice acceptor site will lead to loss of nine amino acids in the 5′ part of exon 8 followed by disturbed reading frame but no generation of a premature stop codon. **c** Sashimi plot visualizing aberrant splicing variants in a sample with a *TP53* mutation in the splice acceptor site of intron 6 compared with a nonmatched normal colonic mucosa sample. The transcript variant using a cryptic splice acceptor site located 49 basepairs upstream of the canonical splice site is identical with the p53ψ isoform, which is truncated due to the introduction of a premature stop codon. The aberrant transcript due to intron retention contains a premature stop codon in intron 6. Sashimi plots for the remaining samples with *TP53* splice mutations are shown in Supplementary Fig. 1

^a^Samples ordered according to estimated relative expression level of canonical transcripts, as shown in Fig. 2a

^b^Mutations are described according to the Human Genome Variation Society (HGVS) nomenclature and using the NM_000546.4 coding sequence as reference

### Diverse aberrant transcript variants caused by *TP53* splice mutations

RNA sequencing revealed altered splicing patterns corresponding with splice mutations in 12 (75%) of the 16 mutated samples, including exon skipping events, intron retention and usage of cryptic splice acceptor and donor sites not detected in an unmatched normal colonic mucosa sample (Table 1, Fig. 2a–c, Supplementary Fig. 1). Two different aberrant transcript variants per sample, in addition to the canonical splice variant, were identified in six samples (Table 1, Supplementary Fig. 1). Disparate splicing consequences between samples with mutations affecting the same canonical splice site were also observed. Samples 8 and 16 harbored base substitutions one and two nucleotides upstream of exon 8, respectively, but only the former exhibited aberrant splicing in this region (Fig. 2b). Furthermore, the estimated expression levels of the aberrant transcript variant(s) relative to the canonical splicing isoform per sample ranged from 7 to 88%, with a median of 31% (Fig. 2a, Supplementary Table S2). Collectively, this shows diverse effects of splice mutations on expressed transcript structures.

Nonetheless, most of the aberrant transcript variants (*N* = 11, 85%) introduced a premature stop codon or altered reading frame, and were predicted to produce truncated TP53, if not degraded by nonsense-mediated decay (Table 1). Among these was the truncated p53ψ isoform which has previously been implicated in cancer^8^. This was expressed in one sample harboring a point mutation two basepairs upstream of exon 7 (c.673–2A, sample 10, Fig. 2c), as a result of activation of a cryptic splice acceptor site located 49 basepairs upstream of the canonical splice site in intron 6. This separation-of-function isoform has been shown to have poor prognostic associations and to induce epithelial to mesenchymal transition (EMT) in a transcriptionally independent manner^8^.

### *TP53* splice mutations lead to reduced expression of TP53 target genes

**Fig. 3:**
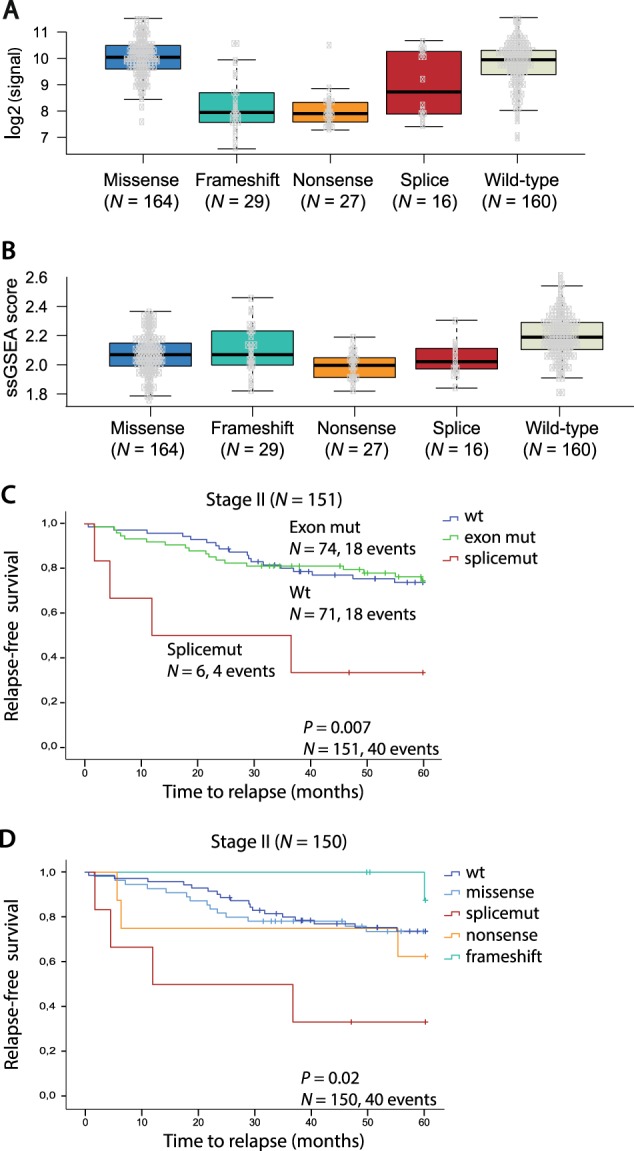
Transcriptional consequences and prognostic associations of *TP53* splice mutations. **a** *TP53* gene expression levels according to mutation type. Due to small sample sizes, in frame indels and silent mutations were not included. **b** *TP53* signature score according to mutation type. Due to small sample sizes, in frame indels and silent mutations were not included. ssGSEA single-sample gene set enrichment analysis. **c** Kaplan−Meier survival curve showing 5-year relapse-free survival (RFS) for patients with *TP53* wt, exon mutations and splice mutations in CRC stage II. **d** 5-year RFS according to *TP53* mutation type in CRC stage II. Due to small sample size, in frame indels and silent mutations (*N* *=* 1) were not included

To analyze the downstream transcriptional consequences of *TP53* splice mutations relative to exon mutations, *TP53* gene expression was compared according to mutation type. Samples with missense mutations had the highest *TP53* expression levels, similar to the expression in tumors with *TP53* wild type (Fig. 3a). Samples with frameshift and nonsense mutations had a significant reduction in the expression of *TP53* (*P* < 0.001), likely because several of these transcripts are eliminated by nonsense-mediated mRNA decay. Samples with splice mutations had a distinct intra-group dichotomy of high and low *TP53* expression, but this was not related to the expression of truncating isoforms. However, differential gene expression analysis showed that both tumors with exon mutations (irrespective of the type) and splice mutations had downregulation of known TP53 transcriptional targets in comparison with wild-type tumors, including *MDM2*, *FAS, HSPA4L* and *SPATA18* among the top five differentially expressed genes (Supplementary Table 3a)^9^. No genes were differentially expressed between the two mutation groups. Furthermore, to compare TP53 pathway activity, a single-sample gene set expression enrichment score was calculated for a set of 200 genes involved in the pathway^10^. All mutation types were associated with significantly lower *TP53* signature scores than wild-type samples (*P* < 0.01), with only minor differences among the mutation types (Fig. 3b, Supplementary Table 3b). Accordingly, the downstream transcriptional consequences of *TP53* splice mutations were similar to those of exon mutations.

No significant differences between *TP53* splice and exon mutations with respect to clinicopathological and molecular associations were found (Supplementary Table 4) and neither splice- nor exon-mutations were associated with survival across all stages (Supplementary Fig. 2a). However, stratification according to TNM stage showed that patients with *TP53* splice mutations in stage II had worse 5-year RFS (*P* *=* 0.007, Fig. 3c) compared to both *TP53* wild-type and exon mutations, although the sample number was low. The same trend was also seen in multivariable analysis comparing splice mutations to exon mutations (RFS: hazard ratio (HR) 4.82 (1.14–20.43); *P* *=* 0.033, Supplementary Fig. 2b). Of note, splice mutations were associated with inferior prognosis in stage II compared with other truncating mutation types, i.e. nonsense and frameshift mutations (*P* *=* 0.02, Fig. 3d), suggesting that the prognostic impact is not due to the loss of full-length TP53 protein per se.

## Discussion

We show that splice mutations account for 6% of all *TP53* mutations in CRC, which is threefold higher than estimated in major databases^4,5^. With respect to expression of corresponding aberrant transcript variants, the splice mutations show great diversity, but on the protein level, the majority are predicted to cause truncated isoforms, in line with the prevailing notion of the effects of such mutations. Nonetheless, all mutation types analyzed in this study, both splice mutations and the different types of exonic mutations, converged on downregulation of both TP53 target genes and signatures of TP53 pathway activity. This, together with the mutual exclusivity to exon mutations, indicate that *TP53* splice mutations have similar biological relevance as alterations in protein-coding sequences.

Intriguingly, *TP53* splice mutations were associated with a poor prognosis in stage II in our patient series. However, the low number of splice mutated samples within each stage and the lack of a detrimental effect of splice mutations across stages warrants cautious interpretation. Still, *TP53* splice mutations have previously been shown to be enriched in metastatic CRC, an association not seen for *TP53* mutations in general^7^. This could conceivably be explained by the generation of novel transcript variants with distinct biological properties beyond their direct effects on the TP53 network, and a propensity for relapse and metastasis, as described for p53ψ and other truncating mutations in exon 6^7^. Our finding of stage-specific inferior prognosis for *TP53* splice mutations compared with other truncating mutations supports the notion of these mutations having biological consequences beyond the loss of wild-type TP53 activity. Studies on non-small cell lung cancer have shown that mutations in the splice acceptor site of intron 6 generate the prometastatic isoform p53ψ, conveying poor prognostic properties. The EMT-inducing properties of this isoform can be inhibited through targeting its downstream effector Cyclophilin D by drugs such as cyclosporine A^8^. To our knowledge, this is the first study to detect the presence and underlying genesis of this potentially prognostic and druggable transcript variant in CRC. We cannot rule out distinct oncogenic properties of the other splice mutations detected in this study.

The lack of RNA sequencing data and *TP53* transcript variant analysis from tumors with nonsplice mutations is a potential limitation of our study. Pan-cancer analysis has shown that synonymous and missense exonic mutations can cause aberrant splicing of tumor suppressor genes in general and most frequently in *TP53*. This occurs most commonly through mutations at residues adjacent to splice junctions^11,12^. Our study cannot rule out such splice-altering exonic mutations, but only two mutations in such residues were detected. This suggests only a very limited subset of splice-altering synonymous and missense mutations leading to truncated TP53 protein with distinct impact on TP53 pathway activity and clinical outcome.

In conclusion, splice mutations account for a 6% subgroup of *TP53*-mutated CRCs, which are mutually exclusive to exon mutations, cause aberrant transcript variant expression, downregulation of downstream target gene expression and pathway activity, and may identify high-risk stage II patients.

## Material and methods

### Patient material

A total of 401 fresh-frozen primary CRCs from a population-representative series of patients operated for CRC stage I−IV at Oslo University Hospital, Norway between 2005 and 2014 were included. DNA extraction was performed as previously described^13,14^. Comprehensive clinicopathological data were prospectively registered for all patients. The research conformed to the Helsinki Declaration and was approved by the Regional Committee for Medical and Health Research Ethics (REC number 1.2005.1629). Written consent was obtained from all patients. The research biobanks have been registered according to national legislation.

### Mutation analyses, microsatellite instability status and CMS classification

*TP53* mutation status was assessed in all 401 samples using Sanger sequencing of the entire coding region (exons 2–11), as well as the first ten and last ten nucleotides of each intron. Splice mutations were defined as any mutation affecting any of these intronic regions, based on the finding that intron mutations outside the invariant AG and GT dinucleotides in the consensus splice acceptor (SA) and splice donor (SD) site, respectively, may lead to missplicing^15^. Tumors harboring only synonymous mutations were classified as *TP53* wild-type (wt) in survival analysis. Microsatellite instability (MSI) analysis, consensus molecular subtype (CMS) classification and mutation analyses for *KRAS* (exon 2: codons 12 and 13) and *BRAF* (codon 600) were performed in all samples as previously described^16–19^. *KRAS* exon 3 codon 61 was analyzed in a subset of samples (*N* = 127).

### Differential gene expression and gene set enrichment analysis

All samples have previously been analyzed for gene expression at the exon-level using Affymetrix GeneChip® Human Exon 1.0 ST Array (HuEx, *N* *=* 199)^13,20^ and are available from GEO (GSE24550, GSE29638, GSE69182, GSE79959), or Human Transcriptome Array 2.0 (HTA 2.0, *N* *=* 202) (GSE79959)^21^ (GSE9652)^18^. Differential gene expression analysis and single-sample gene set enrichment analysis were performed using the R packages limma^22^ and GSVA^23^, respectively. The “HALLMARK_P53_PATHWAY” gene set from the Molecular Signatures Database (v5.2)^10^ was used to assess differential TP53 pathway activity among mutation groups. Dunn’s test implemented in the R package dunn.test was used for statistical significance testing. This is a nonparametric post hoc test after Kruskal−Wallis rank sum tests to identify medians that are significantly different between the possible pairs.

### RNA sequencing, alignment and transcript variant analysis

RNA sequencing was performed for all samples harboring *TP53* splice mutations (*N* *=* 16) detected by Sanger sequencing, and one normal sample for comparison. Strand-specific libraries for TruSeq total RNA sequencing (Illumina Inc.) were prepared according to protocol. Sequencing was performed on the Illumina HiSeq 2500 platform (2 × 101 bp, paired end) to an average of 77.4 million read pairs per sample. FastQC was run for quality control, and reads were aligned to the hg38 (HGMF genome_snp_tran) reference using HiSat2 v. 2.0.4 ^24^. Aligned reads were inspected for transcript variants of *TP53* using the Integrative Genome Viewer (IGV). The discovered transcript variants were visualized with the IGV built-in Sashimi plot function^25^. Exon skipping, usage of cryptic splice sites and canonical splicing in the relevant *TP53* splice regions were detected and quantified by the Sashimi plot estimates, further described in legend to Fig. 2b. To avoid false positives due to sequencing or alignment artifacts, only aberrant transcript variants detected in ≥5 reads and accounting for ≥5% of all detected relevant reads in the sample are considered. As intron retention is not quantified by the Sashimi plots, we used the IRFinder algorithm to detect and quantify this class of splice variants^26^. Only events detected by the IRFinder algorithm with default settings were considered^26^. The threshold for calling intron retention was set at a minimum of 10% of transcripts retaining the intron (IR ratio > 0.1) and with a minimum coverage of three reads for each base pair of the intron, after excluding nonmeasurable intronic regions, as recommended by the authors. The expression levels for intron retention events are reported as the median depth of the relevant intronic region, given as output by IRFinder. Intron 4 of *TP53* could not be evaluated by IRFinder, due to exclusion of low complexity regions or overlapping feature annotations preventing unique mapping of reads. For samples with splice mutations affecting this region, evidence of intron retention was visually analyzed in the Sashimi plots and quantified by the Bedtools coverageBed function^27^.

### Statistical analyses

Statistical analyses were performed using the SPSS 21.0 software (SPSS Inc.). Fisher’s exact or Spearman correlation tests were applied when appropriate to evaluate associations between categorical variables. Five-year overall survival (OS) and 5-year relapse-free survival (RFS) plots were generated by the Kaplan–Meier method and the log-rank test was used to compare plots. The Cox proportional hazards model was used for univariable and multivariable analyses to assess the independence of prognostic factors. RFS and OS were calculated with date of surgery as starting point. All tests are two-tailed and *P* values < 0.05 were considered significant.

## Supplementary information

Supplementary Information
