# Supplementary material for: Transcriptional and functional consequences of TP53 splice mutations in colorectal cancer
Source: Oncogenesis. 2019 May 15;8(6):35. doi: 10.1038/s41389-019-0141-3 (PMC6520361; doi:10.1038/s41389-019-0141-3)
Supplement: Supplementary file 1 — Supplementary Information [file 41389_2019_141_MOESM1_ESM.docx]

# Supplementary Tables

### Supplementary Table 1. *TP53* splice mutations: description in the IARC database

All these mutations were in microsatellite stable colorectal cancers for which no mutations were detected within exonic sequences of *TP53.*

| **Sample no.** | ***TP53* splice mutation** | **c_description^a^** | **Described in IARC^b^** | **CRC in IARC^b^** |
| --- | --- | --- | --- | --- |
| 16 | Exon 8 -2 (SA), A>G | c.783-2A>G | yes | no |
| 2 | Exon 5 -1 (SA), G>A | c.376-1G>A | yes | no |
| 9 | Exon 4, +5, G>A | c.375+5G>A | yes | no |
| 5 | Exon 9 -2 (SA), A>G | c.920-2A>G | yes | yes |
| 15 | Exon 7 +3, ins 6 bp^c^ | c.782+3_782+4ins6 | no | no |
| 8 | Exon 8 -1 (SA), G>T | c.783-1G>T | yes | no |
| 13 | Exon 5 +1 (SD), del G | c.559+1del1 | yes | yes |
| 1 | Exon 3 +1 (SD), G>A | c.96+1G>A | yes | no |
| 7 | Exon 5 -1 (SA), G>T | c.376-1G>T | yes | no |
| 4 | Exon 5 - 1 (SA), G>A | c.376-1G>A | yes | no |
| 11 | Exon 5 +1 (SD), G>A | c.559+1G>A | yes | yes |
| 3 | Exon 7 +1 (SD), G>A | c.782+1G>A | yes | yes |
| 12 | Exon 6 - 1 (SA), G>A | c.560-1G>A | yes | yes |
| 10 | Exon 7 -2 (SA), A>T | c.673-2A>T | yes | yes |
| 14 | Exon 6 -9 (SA), del 14 bp^d^ | c.560-9_564del14 | yes^e^ | yes^f^ |
| 6 | Exon 8 +1 (SD), G>A | c.919+1G>A | yes | no |

^a^ Mutation nomenclature according to HGVS standards and using the NM_000546.4 coding sequence as reference.

^b^ Designated 'yes' if mutation of any kind at that position is described in the International Agency for Research on cancer (IARC) TP53 database across cancer types (left column) or in CRC (right column).

^c^ Duplication of last two basepairs (bp) of exon 7 and first four bp of intron 7, which is inserted four bp downstream of exon 7, hence the consensus donor site is preserved in addition to the introduction of a duplicated donor site downstream.

^d^ Deletion of 14 bp starting 9 bp 5' direction of exon 6, i.e. includes 9 bp of intron 5 and 5 bp of exon 6, and deletes the consensus acceptor site

^e^ Deletions overlapping, but not identical, with this deletion is described

^f^ Mutations affecting this splice area are described in CRC in IARC but no indels

SD: consensus splice donor site, SA: consensus splice acceptor site

### Supplementary Table 2. Estimated relative expression levels of aberrant transcript variants

| **Sample no.** | ***TP53* splice mutation** | **Alternative transcript variant(s)** | **Expression level of alternative transcript(s)^a^** | **IR ratio^b^** |
| --- | --- | --- | --- | --- |
| 1 | Exon 3 +1 (SD), G>A | Exon 3 skipping | 118/134 (88%) | 0.025 |
| 9 | Exon 4 +5, G>A | Intron 4 retention | 39/83 (47%) | N.A^c^ |
| 2 | Exon 5 -1 (SA), G>A | Cryptic SA exon 5 | 69/79 (87%) | N.A |
| 7 | Exon 5 -1 (SA), G>T | Cryptic SA exon 5 | 14/27 (52%) | N.A |
| 4 | Exon 5 -1 (SA), G>A | Cryptic SA exon 5 | 60/72 (83%) | N.A |
| 13 | Exon 5 +1 (SD), del G | Not present | N/A | 0.069 |
| 11 | Exon 5 +1 (SD), G>A | I) Intron 5 retention II) Cryptic SD exon 5 | I) 4/21 (19%) II) 5/21 (24%) | 0.235 |
| 14 | Exon 6 -9 (SA), del 14 bp | Not present | N/A | 0.05 |
| 12 | Exon 6 -1 (SA), G>A | I) Intron 5 retention II) Exon 6 skipping | I) 6/52 (12%) II) 13/52 (25%) | 0.159 |
| 10 | Exon 7 -2 (SA), A>T | I) Intron 6 retention | I) 8/63 (13%)^d^ | 0.214 |
|  |  | II) Cryptic SA intron 6 | II) 19/63 (30%) |  |
| 3 | Exon 7 +1 (SD), G>A | Intron 7 retention | 337/395 (85%) | 0.916 |
| 15 | Exon 7 +3 , ins 6 bp | Not present | N/A | 0.009 |
| 16 | Exon 8 -2 (SA), A>G | Not present | N/A | 0.002 |
| 8 | Exon 8 -1 (SA), G>T | I) Intron 7 retention II) Cryptic SA exon 8 | I) 106/254 (42%) II) 20/254 (8%) | 0.512 |
| 6 | Exon 8 +1 (SD), G>A | I) Intron 8 retention II) Exon 8 skipping | I) 98/203 (48%) II) 15/203 (7%) | 0.549 |
| 5 | Exon 9 -2 (SA) , A>G | I) Intron 8 retention II) Exon 9 skipping | I) 11/36 (31%) II) 11/36 (31%) | 0.403 |

^a^ Ratio of aberrant transcripts reads to all other transcript reads (including canonical and other aberrant transcripts) in the relevant genomic sequence. Expression levels of canonical transcripts and transcripts exhibiting exon skipping and cryptic splice site are defined by Sashimi plots, while intron retention values are median depth of the intronic region as measured by IRFinder.

^b^ Output from the IRFinder algorithm. Measures the ratio of transcripts with retained intron relative to canonical splicing. The ratio does not include other aberrant splicing events, such as cryptic splice sites and exon skipping.

^c^ Intron 4 of *TP53* is not analyzed in IRFinder due to the exclusion of low complexity regions preventing unique mapping of reads. In the four samples (samples 2, 4, 7 and 9) with splice mutations potentially affecting this region, evidence of intron retention was visually analyzed in the Sashimi plots and quantified by Bedtools.

### Supplementary Table 3. Top ten differentially expressed genes between *TP53* wt and *TP53* mutated colorectal cancers

#### A Top ten differentially expressed genes between TP53 wt and exon mutated and splice mutated tumors, respectively. Ranked according to significance level.

| **Comparison** | **Symbol** | **log Fold Change^a^** | **Average Expression** | **t** | **P value** | **Adjusted P value^b^** |
| --- | --- | --- | --- | --- | --- | --- |
| **wt - exonmut** | *MDM2* | 0.975 | 9.827 | 16.099 | 2.62e-45 | 4.19e-41 |
|  | *SPATA18* | 0.881 | 6.130 | 14.474 | 1.78e-38 | 1.42e-34 |
|  | *FAS* | 0.780 | 7.592 | 12.167 | 3.40e-29 | 1.82e-25 |
|  | *DDB2* | 0.577 | 7.815 | 12.038 | 1.08e-28 | 4.33e-25 |
|  | *HSPA4L* | 1.654 | 7.293 | 10.911 | 1.93e-24 | 5.57e-21 |
|  | *DDX27* | -0.789 | 9.481 | -10.902 | 2.09e-24 | 5.57e-21 |
|  | *ZMAT3* | 0.607 | 7.566 | 10.745 | 7.88e-24 | 1.80e-20 |
|  | *ACOT8* | -0.466 | 7.202 | -10.592 | 2.85e-23 | 5.69e-20 |
|  | *NELFCD* | -0.722 | 9.933 | -10.360 | 1.95e-22 | 3.46e-19 |
|  | *RAB27B* | 1.457 | 7.536 | 10.138 | 1.20e-21 | 1.91e-18 |
|  |  |  |  |  |  |  |
| **wt - splicemut** | *HSPA4L* | 2.690 | 7.293 | 6.985 | 1.20e-11 | 1.91e-07 |
|  | *MDM2* | 0.971 | 9.827 | 6.310 | 7.41e-10 | 5.93e-06 |
|  | *FAS* | 1.012 | 7.592 | 6.221 | 1.25e-09 | 6.64e-06 |
|  | *DDB2* | 0.732 | 7.815 | 6.008 | 4.22e-09 | 1.59e-05 |
|  | *SPATA18* | 0.924 | 6.130 | 5.980 | 4.96e-09 | 1.59e-05 |
|  | *SCAMP5* | -0.669 | 5.698 | -5.867 | 9.29e-09 | 2.15e-05 |
|  | *PLCG1* | -0.803 | 8.517 | -5.866 | 9.39e-09 | 2.15e-05 |
|  | *ZGPAT* | -0.377 | 7.816 | -5.805 | 1.31e-08 | 2.21e-05 |
|  | *XPNPEP2* | -1.403 | 5.953 | -5.803 | 1.33e-08 | 2.21e-05 |
|  | *ACOT8* | -0.648 | 7.202 | -5.795 | 1.38e-08 | 2.21e-05 |

^a^ Positive and negative numbers indicate down- and upregulation, respectively, in samples with *TP53* mutations compared to wild-type.

^b^ FDR adjusted P values.

#### B Comparison of TP53 signature scores according to mutation type.

| **Comparison** | **Median diff** | **P adjusted** |
| --- | --- | --- |
| **wt-frameshift** | 0.120 | **0.008** |
| **wt-missense** | 0.121 | **5.29e-12** |
| **wt-nonsense** | 0.193 | **9.47e-11** |
| **wt-splice** | 0.168 | **0.0002** |
| **missense-frameshift** | 0.000 | 1 |
| **nonsense-frameshift** | -0.073 | **0.03** |
| **nonsense-missense** | -0.072 | **0.02** |
| **splice-frameshift** | -0.048 | 0.78 |
| **splice-missense** | -0.048 | 1 |
| **splice-nonsense** | 0.025 | 1 |

Dunn’s Test for difference in TP53 single sample Gene Set Enrichment Analysis (ssGSEA) score between *TP53* groups. Bonferroni adjusted P values.

### Supplementary Table 4. Association between *TP53* mutation status and clinicopathological and molecular characteristics

|  |  | **wt** | **exon mut** | **splicemut** | **P values^a^** | | | |
| --- | --- | --- | --- | --- | --- | --- | --- | --- |
| **Characteristics** |  | **No. (%)** | **No. (%)** | **No. (%)** | **splice vs exonmut** | **splice vs wt** | **wt vs exonmut** | **wt vs total mut** |
| **Age (years)** | ≤ 70 | 77 (48) | 119 (53) | 7 (44) | 0.61 | 0.8 | 0.41 | 0.48 |
|  | > 70 | 83 (52) | 106 (47) | 9 (56) |  |  |  |  |
| **Gender** | Male | 67 (42) | 117 (52) | 11 (69) | 0.3 | 0.06 | 0.06 | **0.032** |
|  | Female | 93 (58) | 108 (48) | 5 (31) |  |  |  |  |
| **MSI status** | MSS | 98 (62) | 206 (95) | 16 (100) | 1 | **0.001** | **<0.001** | **<0.001** |
|  | MSI | 60 (38) | 12 (6) | 0 |  |  |  |  |
| ***KRAS*** | wt | 108 (68) | 153 (68) | 12 (75) | 0.78 | 0.78 | 1 | 0.91 |
|  | mut | 52 (33) | 72 (32) | 4 (25) |  |  |  |  |
| ***BRAF*** | wt | 113 (71) | 207 (92) | 13 (81) | 0.15 | 0.56 | **<0.001** | **<0.001** |
|  | mut | 47 (29) | 18 (8) | 3 (19) |  |  |  |  |
| **CMS** | CMS1 | 44 (36) | 17 (10) | 1 (8) | 0.97 | **0.024** | **<0.001** | **<0.001** |
|  | CMS2 | 29 (23) | 101 (56) | 8 (62) |  |  |  |  |
|  | CMS3 | 29 (23) | 23 (13) | 2 (15) |  |  |  |  |
|  | CMS4 | 22 (18) | 38 (21) | 2 (15) |  |  |  |  |
| **Location** | Right | 94 (59) | 69 (31) | 8 (50) | 0.39 | 0.66 | **<0.001** | **<0.001** |
|  | Left | 27 (17) | 87 (39) | 4 (25) |  |  |  |  |
|  | Rectum | 38 (24) | 67 (30) | 4 (25) |  |  |  |  |
|  | Synchronous | 1 (1) | 2 (1) | 0 |  |  |  |  |
| **TNM stage^b^** | 1 | 31 (19) | 52 (23) | 1 (6) | 0.133 | 0.051 | 0.403 | 0.26 |
|  | 2 | 71 (44) | 74 (33) | 6 (38) |  |  |  |  |
|  | 3 | 42 (26) | 66 (29) | 5 (31) |  |  |  |  |
|  | 4 | 16 (10) | 33 (15) | 4 (25) |  |  |  |  |
| **Differentiation^b,c^** | High | 5 (3) | 4 (2) | 0 | 0.46 | 0.60 | **0.003** | **0.003** |
|  | Medium | 117 (73) | 197 (88) | 14 (88) |  |  |  |  |
|  | Low | 33 (21) | 18 (8) | 2 (13) |  |  |  |  |
| **Relapse after R0** | No relapse | 142 (89) | 190 (84) | 14 (88) | 1 | 1 | 0.29 | 0.3 |
|  | Relapse | 18 (11) | 35 (16) | 2 (13) |  |  |  |  |

^a^ Fisher's exact two-tailed unless otherwise stated. Significant P values in bold.

^b^ P values from Spearman Correlation test

^c^ Mucinous and other histologies excluded (n=11)

# Supplementary Figures

### Supplementary Figure 1. p53 transcript variants in splice mutated samples

Sashimi plots visualizing read depth, aberrant splicing, and transcript structure generated from RNA sequencing data from splice mutated samples compared with a non-matched normal colonic tissue sample. Tumor samples with mutations in the same splice regions are presented in the same figure for comparison of their transcriptional consequences. Reading frame right to left. Reads spanning each junction are represented by arcs linking exonic regions, and each arc is labelled with the average number of reads spanning that particular junction. Arcs representing aberrant splicing events are coloured in orange. Heights of bars show coverage values at genomic locations. Position of splice mutation marked with dashed line. Exons and introns according to canonical *TP53* are shaded with different background colours, parts of exons left out due to aberrant splicing are shaded in pink. Schematic visualization of transcript variants depicted below for some samples, with coding sequences in light grey, disturbed reading frame in dark grey, non-coding sequences in black excluded exonic sequences in red.

**A**

**
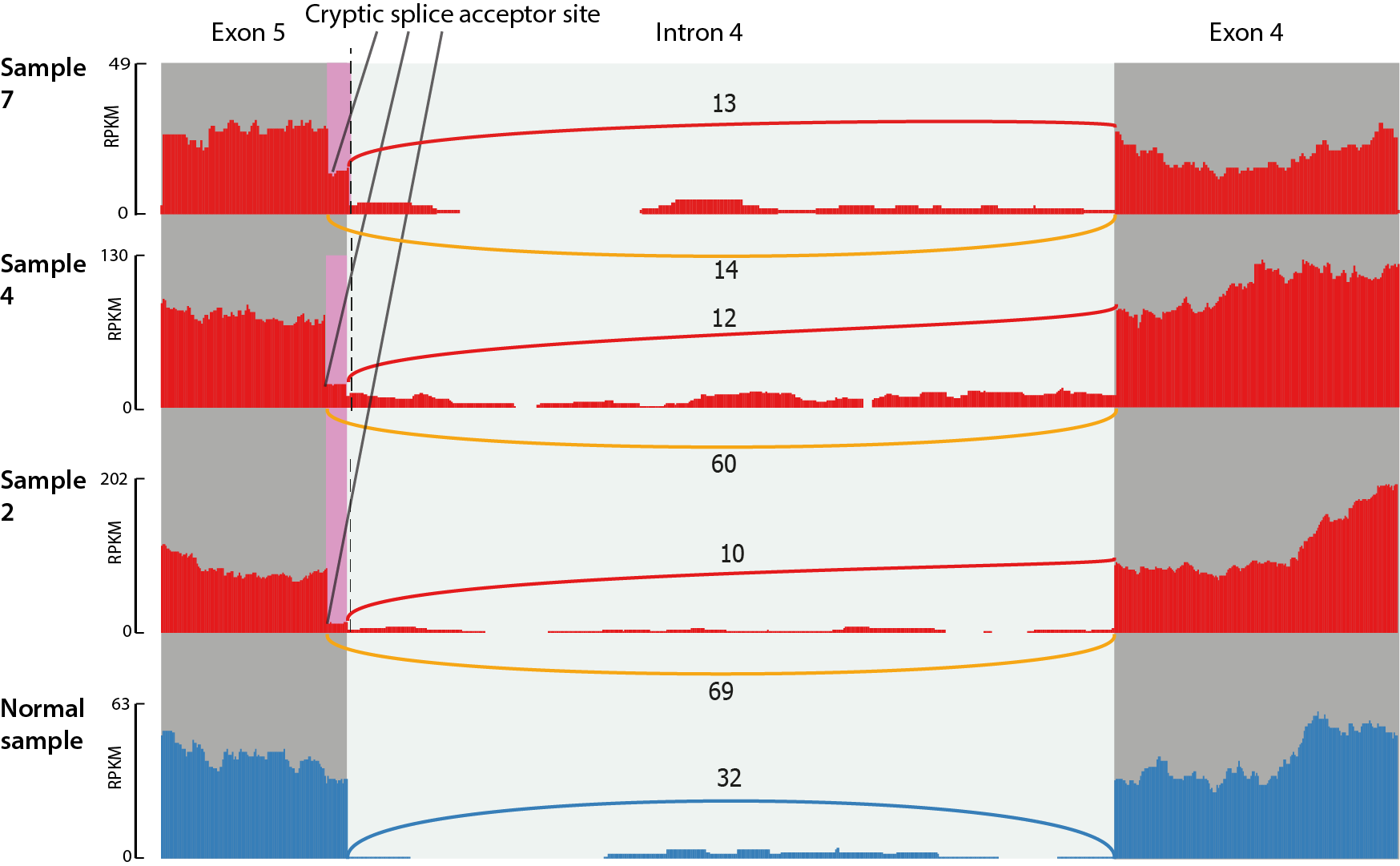
**


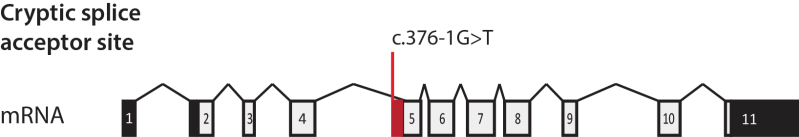


Sashimi plots from three tumor samples harbouring mutation in splice acceptor site of intron 4. All three samples exhibit reads spanning from exon 4 to a cryptic acceptor site 21 basepairs into exon 5. The aberrant transcripts keep the reading frame intact and is predicted to encode a protein with the seven first amino acids of exon 5 missing, corresponding to codons 126-132 located in the loop-sheet-helix (LSH) motif of the DNA-binding domain, but otherwise unaltered.

**B**


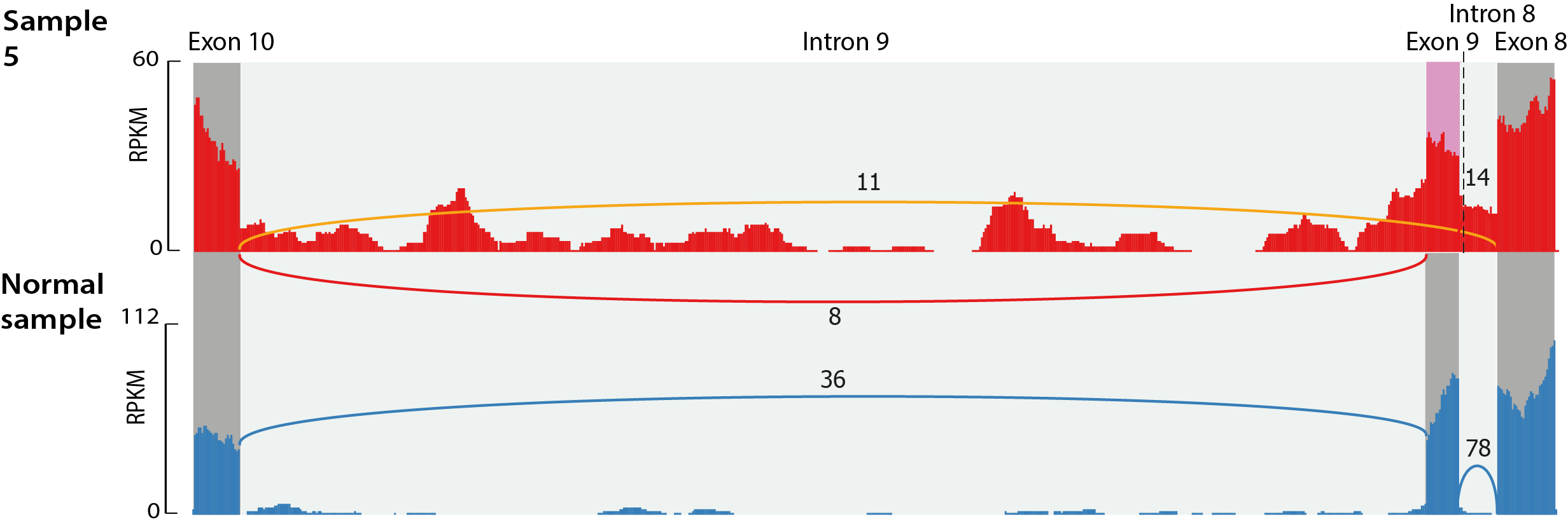


Sashimi plot from tumor sample with mutation in splice acceptor site of intron 8. Eleven junction reads span transcript variants excluding exon 9, while fourteen reads span transcripts using canonical splicing between exon 8 and exon 9. Eleven reads retain intron 8 (median depth of intron 8 as measured by IRFinder). The transcript variant retaining intron 8 is predicted to generate a premature stop codon from intron 8. Skipping of exon 9 will lead to the transcript being out-of-frame from the 5’ end of exon 10 with generation of a premature stop codon.

**C**

**
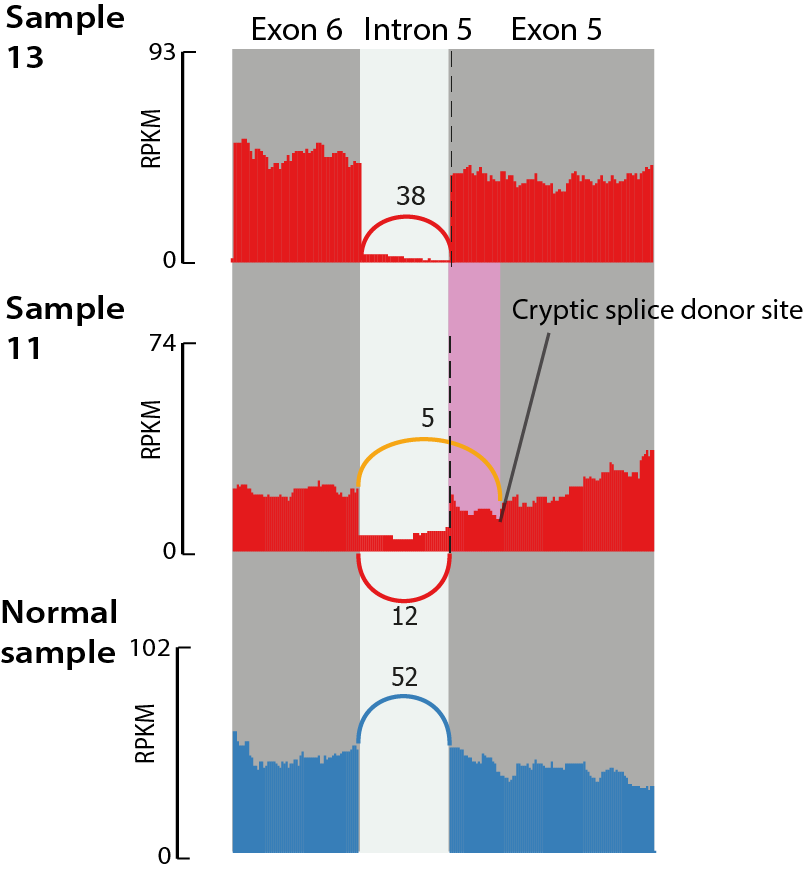
**

Sashimi plots from tumor samples with base substitution (sample no. 11) and single base deletion (sample no. 13) in splice donor site of intron 5. In sample no. 11 five junction reads span transcripts using a cryptic donor site located in the 3’ part of exon 5 and missing 46 basepairs of the exon. Four reads retain intron 5 (median depth of intron 5 as measured by IRFinder), while twelve junction reads span the canonical splice variant between exon 5 and exon 6. The polypeptide produced from the transcript variant missing 46 basepairs of exon 5 is, in addition to the absence of 15 amino acids at the 3’ part of exon 5, expected to produce a premature stop codon in exon 7. The transcript variant retaining intron 5 is predicted to have a premature stop codon located 24 trinucleotides from the canonical donor site of exon 5. In sample no. 13 all junction reads between exon 5 and exon 6 span the canonical splice sites.

**D**


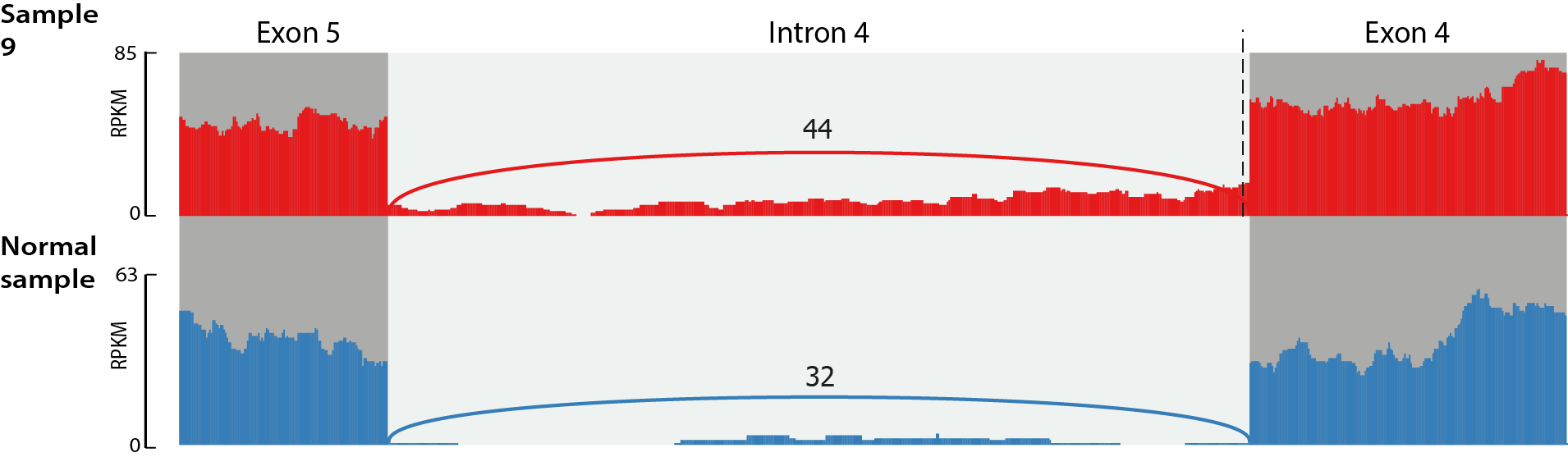


Sashimi plot from a tumor sample with a mutation located five nucleotides downstream of exon 4, i.e. not located in a canonical splice site. In the tumour sample 39 reads retain intron 4 (number of reads overlapping with intron 4 from bedtools coverage). The transcript variant retaining intron 4 is predicted to generate a premature stop codon from intron 4.

**E**

**
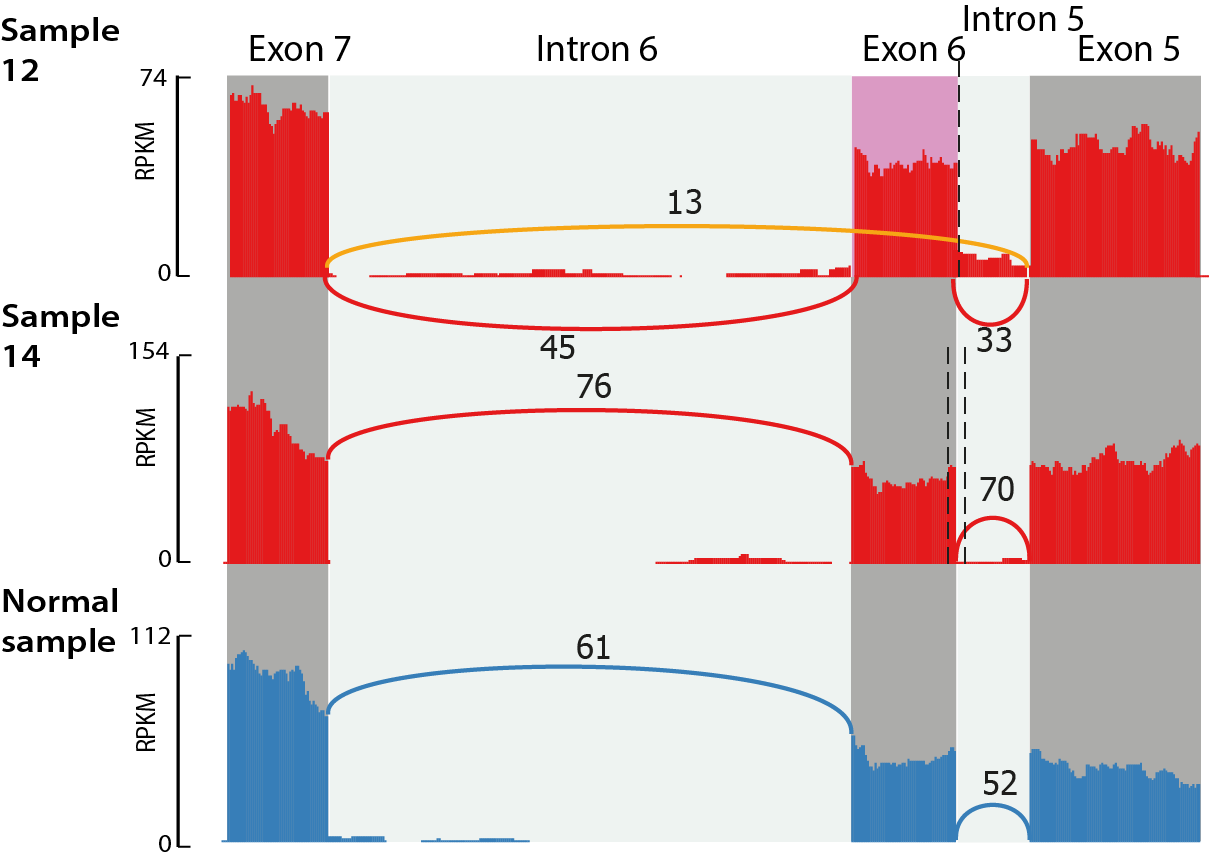
**

Sashimi plots from tumor samples with point mutation in canonical splice acceptor site of intron 5 (sample no. 12) and a deletion of 14 basepairs spanning the intron 5/exon 6 boundary (sample no. 14). In sample no. 12 thirteen junction reads span transcript variants excluding exon 6 and six reads retain intron 5 (median depth of intron 5 as measured by IRFinder), while 33 reads span transcripts using canonical splicing between exon 5 and exon 6. Both aberrant transcripts generate a premature stop codon. In sample no. 14, only transcripts spanning the canonical splicing regions are evident.

**F**


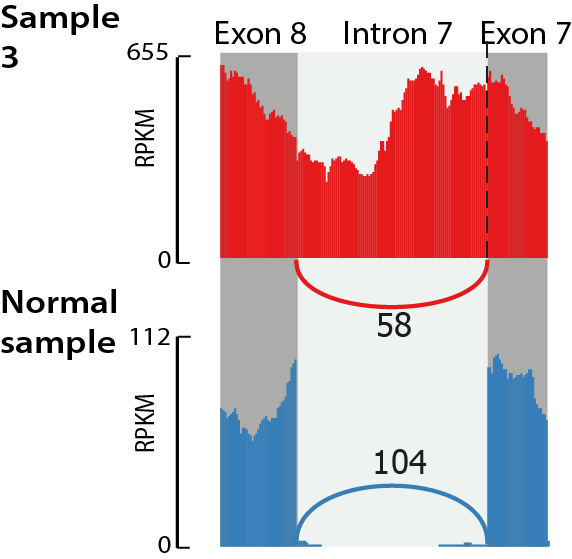


Sashimi plot from a sample with a mutation in splice donor site of intron 7. 337 reads retain intron 7 (median depth of intron 7 as measured by IRFinder), while 58 reads span transcripts using the canonical splice sites between exon 7 and 8. The transcript variant retaining intron 7 is predicted to generate a premature stop codon. Tumor sample no. 15 (Sashimi plot not shown due to low graphical quality) also harboured a mutation in the same splicing region, specifically a duplication of six basepairs spanning the exon 7/intron 7 boundary. In this sample no evidence of aberrant transcript variants was seen.

**G**


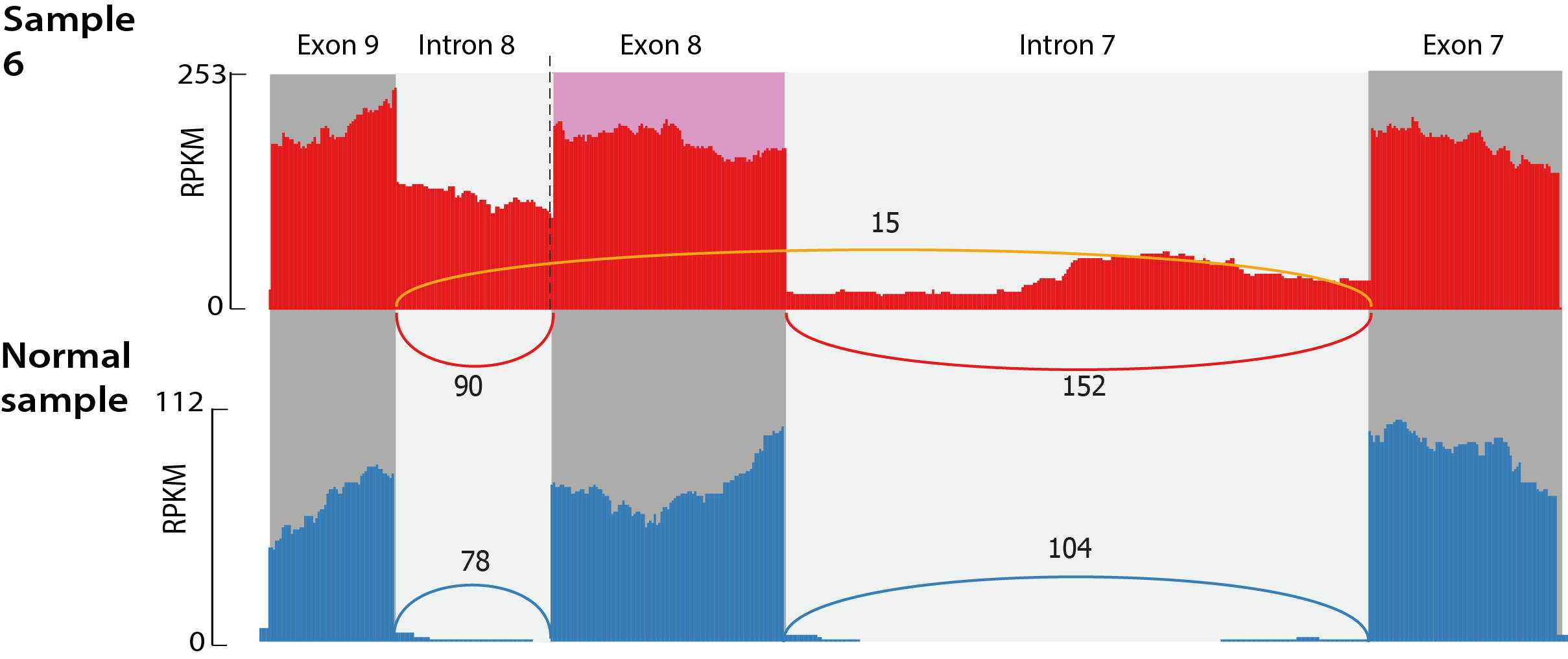


Sashimi plot generated from a tumor sample with mutation in the canonical splice donor site of intron 8. In the tumour sample 15 junction reads span transcripts skipping exon 8, while 98 reads retain intron 8 (median depth of intron 8 as measured by IRFinder). Both aberrant transcript variants are expected to introduce premature stop codons.

**H**


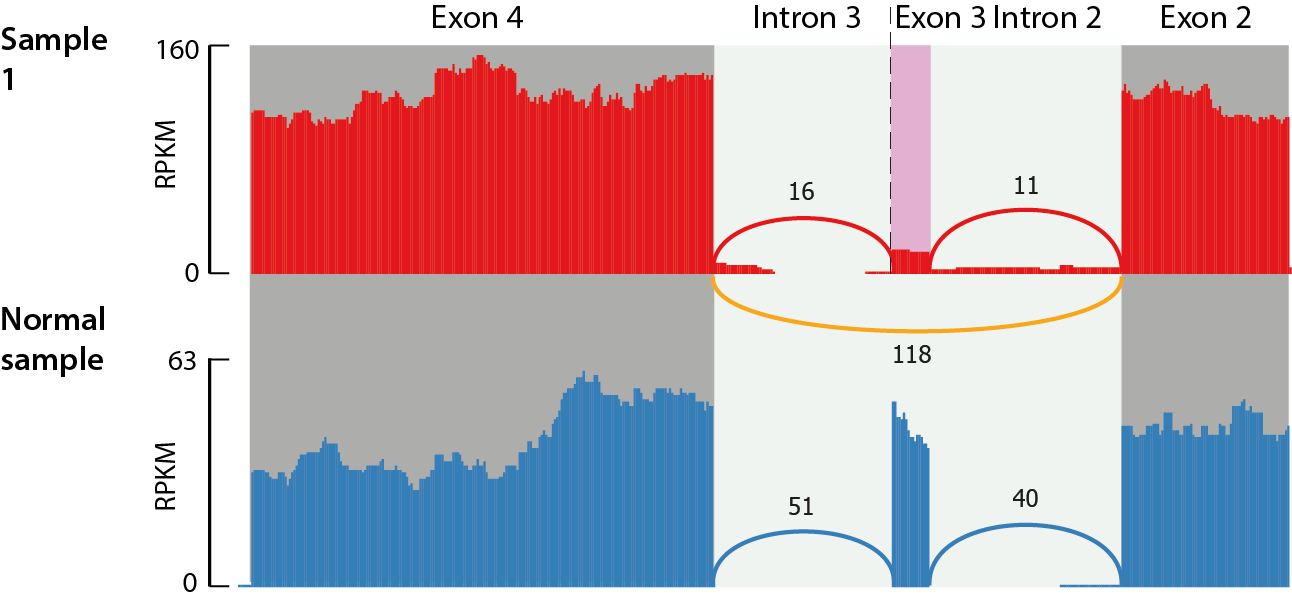


Tumor sample with mutation in splice donor site of intron 3. The aberrant transcript due to exon skipping contains a premature stop codon in exon 4. The transcript variant will introduces a premature stop codon in exon 4.

### Supplementary Figure 2. Prognostic impact of *TP53* exon and splice mutations

Kaplan-Meier survival curves showing A) 5 year overall survival (OS) for patients with *TP53* wt, exon mutations and splice mutations in CRC, across all stages , B) 5 year relapse free survival (RFS) for patients with *TP53* wt, exon mutations and splice mutations in CRC stage II. Multivariate analysis incorporates age, gender, location, MSI status, molecular subtype and mutation status of *KRAS* and *BRAF*.

**A**


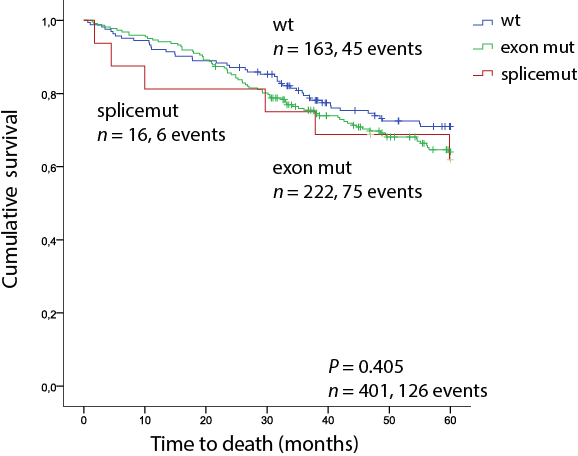


**Univariate analysis:**

Splice mut vs wt: HR 1.42 (0.61-3.32); *P* = 0.422, Cox Regression model

Splice mut vs exon mut: HR 1.11 (0.48-2.55); *P* = 0.804

Exon mut vs wt: HR 1.27 (0.88-1.83); *P* = 0.211

**B**


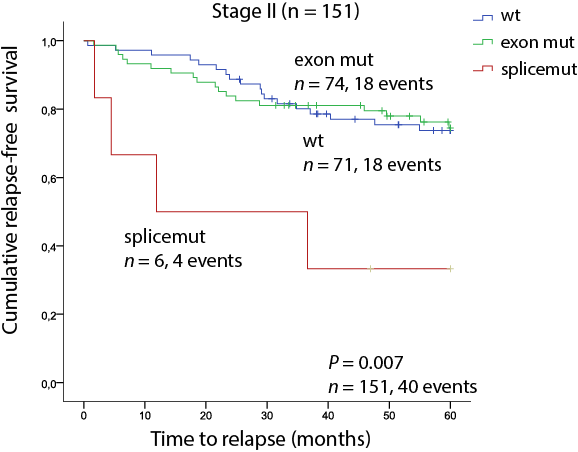


**Univariate analysis:**

Splice mutation vs wt: HR 4.42 (1.49-13.16); *P* = 0.008 Cox Regression model

Splice mutation vs exon mutation: HR 4.52 (1.52-13.46); *P* = 0.007

Exon mutation vs wt: HR 0.99 (0.51-1.90); *P* = 0.968

**Multivariate analysis:**

Splice mutation vs wt: HR 2.08 (0.38-11.37); *P* = 0.399 Cox Regression model

Splice mutation vs exon mutation: HR 4.82 (1.14-20.43); *P* = 0.033

Exon mutation vs wt: HR 0.62 (0.26-1.48); *P* = 0.27
